# Recent advances in the diagnosis and treatment of refractory ocular inflammatory diseases: focus on uveitic macular edema, acute retinal necrosis, and vitreoretinal lymphoma

**DOI:** 10.1007/s10384-025-01310-3

**Published:** 2025-12-10

**Authors:** Atsunobu Takeda, Nobuyo Yawata, Koh-Hei Sonoda

**Affiliations:** 1 Department of Ophthalmology, Faculty of Medicine, Oita University, Yufu, Oita 879-5593 Japan; 2 Department of Ocular Pathology and Imaging Science, Graduate School of Medical Science, Kyushu University, Fukuoka, Japan; 3 Department of Ophthalmology, Graduate School of Medical Science, Kyushu University, Fukuoka, Japan

**Keywords:** Uveitis, Uveitic macular edema, Acute retinal necrosis, Masquerade syndrome, Vitreoretinal lymphoma

## Abstract

Refractory inflammatory ocular diseases—uveitic macular edema (UME), acute retinal necrosis (ARN), and vitreoretinal lymphoma (VRL)—pose significant diagnostic and therapeutic challenges due to their vision- or life-threatening nature and limited treatment options. UME, a leading cause of vision loss in intermediate, posterior, and panuveitis, affects approximately 40% of such cases, particularly in elderly patients and those with prolonged inflammation. Despite its prevalence, effective treatment is still being explored. ARN, caused by herpes viruses such as herpes simplex virus (HSV)-1, HSV-2, and varicella-zoster virus, ranks among the retinal diseases with the poorest visual prognosis; nearly half of patients experience a visual acuity (VA) of ≤ 0.1 within six months. However, a standardized treatment regimen has not yet been established. VRL, one of the ocular malignancies with the lowest overall survival rate, is frequently misdiagnosed as uveitis. Given the diagnostic delay and high frequency (60–85%) of central nervous system involvement—which is often directly life-threatening—early detection and comprehensive systemic management are essential. This review highlights recent advances in the diagnosis, clinical trials, and management of these three challenging ocular inflammatory diseases, emphasizing unmet needs and emerging therapeutic strategies.

## Introduction

Inflammatory ocular diseases are important causes of visual morbidity. Of these many diseases, uveitic macular edema (UME), acute retinal necrosis (ARN), and vitreoretinal lymphoma (VRL) are highly refractory and often result in vision- or life-threatening outcomes.

UME is the most common cause of vision loss in intermediate, posterior, and panuveitis, occurring in nearly 40% of these patients [1]. Risk factors include older age, long-standing inflammation, and the presence of an epiretinal membrane (ERM) [2, 3]. Corticosteroids and conventional immunomodulators are commonly used as treatment; however, adverse effects and limited efficacy restrict long-term outcomes [4]. Biologic agents, including anti–tumor necrosis factor (TNF) therapies such as adalimumab and infliximab, as well as tocilizumab, have shown promise [5, 6]; however, no randomized controlled trials of their efficacy have been conducted, and reliable long-term comparative data are lacking.

ARN is a fulminant necrotizing retinitis caused by herpes simplex virus (HSV) or varicella-zoster virus (VZV) [7, 8]. Even with prompt antiviral therapy, almost half of affected eyes decline to severe vision loss within six months [9]. Intravenous acyclovir is widely employed, but no standardized regimen incorporating intravitreal antivirals, adjunctive corticosteroids, or prophylactic vitrectomy has yet been established [10].

VRL is a rare form of primary central nervous system lymphoma (PCNSL) that commonly masquerades as chronic uveitis [11]. Delayed diagnosis is common, and 60–85% of patients develop central nervous system (CNS) involvement, which largely determines prognosis [12]. Ancillary diagnostic methods, such as interleukin (IL)-10/IL-6 cytokine analysis, detection of monoclonality in B or T cell lymphoma, and gene mutation analysis for *myeloid differentiation factor 88*, are useful in the diagnosis of VRL [13]. Currently, there is no standardized treatment for VRL; therapeutic options include local, systemic, or combined therapies, yet formal international consensus guidelines remain undefined.

These three conditions exemplify the unmet needs in the field of refractory inflammatory ocular diseases (Table 1), with major issues including delayed or difficult diagnosis, lack of standardized therapy, and unsatisfactory treatment outcomes. This review summarizes the recent advances in the diagnosis and management of UME, ARN, and VRL, highlighting ongoing challenges and future directions.

**Table 1:** A list of the unmet needs for UME, ARN, and VRL

Name of diseases	List of unmet needs
Uveitic macular edema	1. Standardized treatment algorithms 2. Heterogeneous therapeutic response 3. Need for reliable biomarkers 4. Refractory and recurrent cases 5. Sustained effective therapy
Acute retinal necrosis	1. Standardized treatment protocols 2. High risk of irreversible vision loss 3. Review of the significance and indications for prophylactic vitrectomy 4. Reliable prognostic biomarkers for visual acuity 5. Need for multicenter collaborative studies
Vitreoretinal lymphoma	1. Standardized care pathways 2. Early and accurate diagnosis 3. Relapse surveillance – lack of subclinical recurrence 4. Reliable prognostic biomarkers 5. Control for CNS dissemination 6. Therapy tolerability in older adults 7. Role of emerging therapeutic agents, such as BTK inhibitors, etc. 8. Need for multicenter, prospective or randomized studies comparing local vs systemic vs combined strategies

*UME* uveitic macular edema, *ARN* acute retinal necrosis, *VRL* vitreoretinal lymphoma, *CNS* central nervous system, *BTK* bruton’s tyrosine kinase

### Uveitic macular edema

Uveitis is an inflammatory disease of the uvea, which comprises the iris, ciliary body, and choroid. It accounts for approximately 10–15% of visual impairment among the working-age population in developed countries [14, 15]. Complications of uveitis that may result in vision loss include cataracts, glaucoma, vitreous opacity, macular edema (ME), and retinal damage secondary to ERM formation. Among these, UME is the most common cause of vision loss, affecting 20–70% of patients with uveitis. Approximately 40% of these patients experience VA of less than 20/60 in at least one eye [2, 16]. Although multiple treatment options have been introduced, VA remains unimproved in approximately one-third of patients with UME [17]. Furthermore, UME is reported to be more prevalent in elderly individuals and those with chronic or long-standing uveitis [2].

According to analyses based on the anatomical classification of uveitis, intermediate uveitis exhibits the highest association with ME, with reported rates ranging from 25% to 70% [2]. Non-infectious uveitis carries a significantly greater risk of ME and visual impairment compared to the infectious and idiopathic forms [18]. A study conducted in Singapore identified ME as the second most frequent complication in infectious and idiopathic uveitis, whereas in non-infectious cases it was the most frequent [19]. In addition, ME is a common and vision-threatening complication in various types of uveitis, including acute retinal necrosis (ARN), birdshot chorioretinopathy, juvenile idiopathic arthritis (JIA), ocular sarcoidosis, and Behçet’s disease [20]. ARN and birdshot chorioretinopathy are associated with particularly high rates of ME, occasionally reaching 100% [16]. ME is especially frequent in JIA-related uveitis, with reported rates exceeding 60% [16, 21, 22]. In ocular sarcoidosis, ME has been documented in approximately 22.6–24% of cases in Japan and 59% of cases in the Netherlands [16, 23, 24]. In Behçet’s disease, ME has been reported in 15% to 63% of patients [16, 25, 26].

#### Diagnostic imaging of uveitic macular edema

**Fig. 1:**
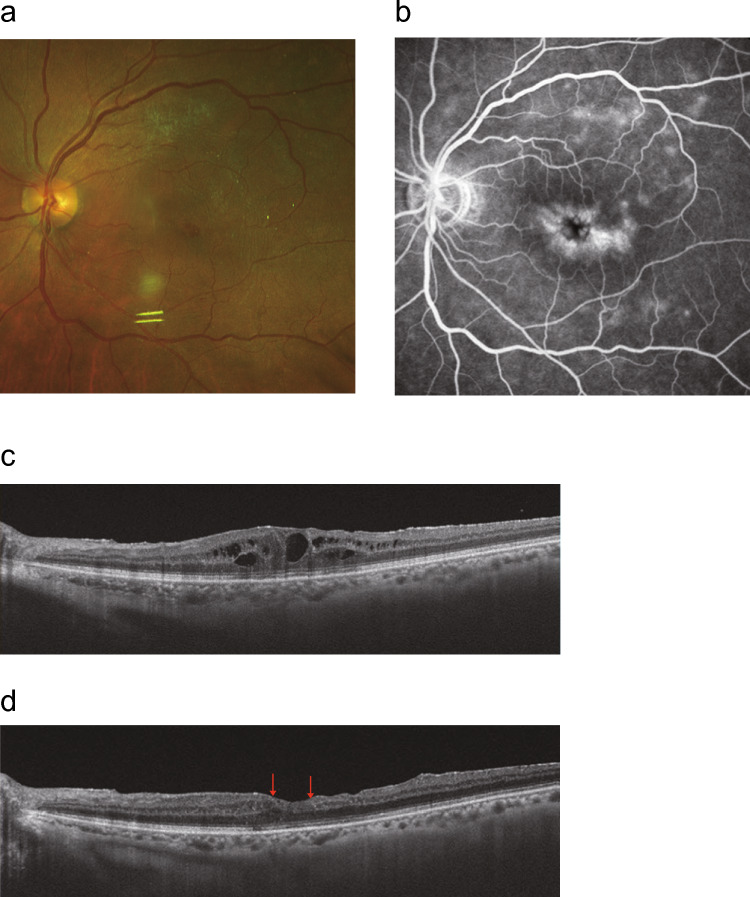
Representative images of the left eye of a patient with uveitic macular edema, secondary to Behçet’s disease. **a** Fundus photograph showing cystoid macular edema. **b** Fluorescein angiography showing diffuse macular hyperfluorescence with late-phase leakage, consistent with cystoid macular edema secondary to intraocular inflammation. **c** Spectral-domain optical coherence tomography (OCT) image before sub-Tenon’s capsule injection of triamcinolone acetonide (STTA) injection, showing multiple cystoid spaces within the inner retina. **d** Spectral-domain OCT image one month after STTA. Macular edema has resolved; however, disorganization of the inner retinal layers (DRIL) is observed between the red arrows

Optical coherence tomography (OCT) and fluorescein fundus angiography are highly sensitive diagnostic tools used for detecting UME [27]. OCT is a noninvasive imaging modality that allows for repeated evaluations and is particularly useful for monitoring treatment response. It can clearly demonstrate various forms of ME, including cystoid macular edema (CME), diffuse ME, and serous retinal detachment (SRD). Among these, CME is the most common, followed by diffuse ME, whereas SRD is the least frequent manifestation [2]. Studies utilizing spectral-domain OCT to assess UME treatment outcomes indicate that disruption of the ellipsoid zone and disorganization of the inner retinal layers (DRIL) are associated with visual impairment (Fig.1). Additionally, in cases with SRD, treatment has been shown to induce remission of UME [28]. SRD is believed to result from disruption of the outer blood–retinal barrier (BRB), dysfunction of the retinal pigment epithelium (RPE) pump, and tractional forces exerted by the Müller cell cone. It is suggested that when the interaction between Müller cells and foveal cone photoreceptors is severely impaired, SRD may not develop, resulting instead in a subtype of ME without SRD [29].

#### Pathogenesis of uveitic macular edema

Intraocular inflammation caused by uveitis induces inflammatory mediators, such as cytokines, which disrupt the BRB [30]. Once the BRB is compromised, plasma components and interstitial fluid leak from blood vessels and accumulate within the retina, leading to the development of ME. Additionally, formation of an ERM can exert mechanical traction on the macula, further contributing to ME [3].

Several studies have investigated inflammatory mediators associated with UME. Valentincic et al. report elevated levels of interleukin (IL)-6 and IL-8 in both the serum and aqueous humor (AH), as well as increased serum TNF-α, in patients with macular edema secondary to intermediate uveitis [31]. In contrast, no significant difference in IL-6 and IL-8 expression was observed in the AH between patients with and without UME [32]. Our group also performed a comprehensive analysis of approximately 30 cytokines in the vitreous humor (VH) of patients with sarcoidosis and Behçet’s disease using a multiplex immunoassay. Notably, the vitreous levels of two factors—soluble interleukin-6 receptor (sIL-6R) and B-cell activating factor belonging to the tumor necrosis factor family (BAFF)—were significantly elevated in patients with UME compared to those without UME [33]. IL-6 has been studied for its effects on the BRBs. It directly promotes the breakdown of the outer BRB and indirectly disrupts the inner BRB by inducing the vascular endothelial growth factor (VEGF) in vascular endothelial cells, which then acts in an autocrine manner [34]. Although IL-6 receptor (IL-6R) expression is limited to specific cell types, the IL-6/sIL-6R complex can mediate signaling in IL-6R–negative but gp130-positive cells due to the ubiquitous expression of gp130—a mechanism known as IL-6/sIL-6R trans-signaling [35]. Tocilizumab (TCZ), an anti-IL-6 agent, has also been shown to inhibit BRB breakdown [34]. Additionally, IL-6 enhances the expression of transforming growth factor-beta derived from activated T lymphocytes, thereby promoting the fibrosis cascade [36]. These findings suggest that IL-6 and sIL-6R contribute not only to BRB disruption but also to the development of ME associated with uveitis, potentially via ERM formation.

#### Treatment of uveitic macular edema

ME requires early intervention, as untreated cases may lead to irreversible visual impairment. However, no standardized treatment for UME has been established. Local treatment with sub-Tenon’s triamcinolone acetonide injection (STTA) is commonly administered. In the chronic phase, when inflammation has subsided but ME persists or recurs, STTA—with or without systemic therapy—should be employed to resolve the condition. If ME does not resolve following the administration of STTA, alternative treatments should be considered. Furthermore, if STTA fails to produce a therapeutic effect, intravitreal injection of triamcinolone acetonide (IVTA)— an off-label use—or systemic administration of corticosteroids, immunosuppressants, or anti–TNF agents may be indicated whenever systemic therapy has not yet been initiated. Local corticosteroids’ administration carries risks such as steroid-induced glaucoma, cataracts, and ocular infections. Systemic corticosteroids therapy may also cause complications, including diabetes, osteoporosis, and infections, which can limit long-term use. Additionally, some patients may develop resistance to steroid therapy over the disease course.

Other intraocular implant formulations providing sustained corticosteroids release have been developed and are in use in Europe and the United States; in Japan, however, these treatments are not covered by insurance. Available implants include Ozurdex® (dexamethasone), which remains effective for approximately six months, and Retisert® (fluocinolone acetonide), which provides sustained action for two to three years [27].

The Multicenter Uveitis Steroid Treatment (MUST) Trial—a prospective study evaluating the treatment of UME with fluocinolone acetonide intraocular implants for ≥ one year—reports that 94% of patients responded to treatment; however, 43% experienced recurrence and required additional therapy with STTA or IVTA [37]. In this study, the presence of an ERM detected on pre-treatment OCT was identified as a risk factor associated with poor resolution of UME. A randomized controlled trial—the PeriOcular versus INTravitreal corticosteroids for the treatment of uveitic macular edema (POINT) study—compared the efficacy of three corticosteroid therapies: STTA, IVTA, and dexamethasone intraocular implants (Table 2). The study also found that ERM, as detected on OCT before treatment initiation, was a risk factor for persistent UME. Both IVTA and dexamethasone intraocular implants significantly improved best-corrected visual acuity (BCVA) and central foveal thickness (CFT) compared to STTA at eight weeks, although they were associated with a modest increase in intraocular pressure [38].

**Table 2:** Comparison of the effects of local therapies for UME in existing randomized controlled trials

Reference, No	Study name	Study type	Total number of UME eyes/Patients	Local therapy	Observation time or mean/median follow-up time	Percentage of CFT resolution to baseline (%) or numbers of complete remission	Rate of nonassigned therapies (%) or numbers of drug withdrawal due to adverse events, insufficient efficacy, or remission of uveitic activity
Thorne JE, et al. [27]	POINT	Pro-spective	235/192	STTA, IVTA, or dexamethasone implant	24 weeks	23, 39, 46 (%)	45, 7, 5 (%)
The Multicenter Uveitis Steroid Treatment Trial Research Group. [37]	MERIT	Pro-spective	225/194	Dexamethasone implant, IVR, or IV-MTX	24 weeks	34, 5, 8 (%)	7, 37, 55 (%)
Barroso-Garcia N, et al. [8]	N/A	Retro-spective	72/49	ADA, IFX, or TCZ	12 months	19/25, 9/15, 6/9	8/25. 8/15, 2/9

*UME* uveitic macular edema, *Pt*. patients, *CFT* central foveal thickness, *MUST* Multicenter Uveitis Steroid Treatment Trial, *RCT* randomized controlled trial, *POINT* PeriOcular versus INTravitreal corticosteroids for the treatment of uveitic macular edema, *STTA* sub-Tenon’s capsule triamcinolone acetonide injection, *IVTA* intravitreal triamcinolone acetonide injection, *IVR* intravitreal ranibizumab injection, *IV*-*MTX* intravitreal methotrexate injection, *N/A* not applicable, *ADA* adalimumab, *IFX* infliximab, *TCZ* tocilizumab

The therapeutic effect of systemic cyclosporine on bilateral UME has been examined in a retrospective study. The underlying conditions included T cell–mediated inflammatory diseases such as multifocal choroiditis, sarcoidosis, Behçet’s disease, and sympathetic ophthalmia. According to the findings, complete resolution of UME in at least one eye was observed in 70.8% of patients at 6 months, 71.4% at 10 years, and 54.2% at 20 years following treatment initiation [39]. Although systemic administration of anti–TNF agents is reported to be effective for UME, Kunimi et al. found that these agents were beneficial in Behçet’s disease but not in sarcoidosis-associated ME, suggesting that therapeutic responses vary depending on the underlying disease [40]. In the U.S. multicenter randomized clinical trial STOP-Uveitis, which evaluated the efficacy of the anti–IL-6 agent tocilizumab for noninfectious uveitis six months after treatment initiation, 43.5% of patients showed improvement in BCVA by two or more lines and a reduction in vitreous opacity [5]. Additionally, central foveal retinal thickness decreased by an average of 117.43 µm. In Europe, a clinical trial of TCZ for UME reports a positive response in all 44 enrolled patients [41]. A retrospective comparison of the therapeutic efficacy of TCZ versus anti–TNF agents in Behçet’s disease–associated UME shows that both agents were effective; however, TCZ did not demonstrate significantly greater efficacy in patients who were refractory to anti–TNF therapy [6]. Similarly, a French multicenter retrospective study comparing TCZ and anti–TNF agents in patients with UME found that the complete remission rate at six months was 21.8% in the anti–TNF-α group, whereas 35.8% of patients treated with TCZ achieved complete remission, suggesting that TCZ is more effective than anti–TNF agents in the treatment of UME [42]. Phase 3 clinical trials, named MEERKAT and SANDCAT, are currently underway to evaluate the efficacy of intravitreal vamikibart (RG6179), an anti–IL-6 agent, for UME.

Studies report that the therapeutic effect of the treatment of UME with the anti–VEGF agent bevacizumab, is transient, and that bevacizumab alone is ineffective in certain cases [43, 44]. The Macular Edema Ranibizumab versus Intravitreal Anti-inflammatory Therapy (MERIT) Trial was a randomized controlled study comparing the efficacy of three intravitreal treatments for persistent or recurrent UME: ranibizumab (IVR), an anti-VEGF agent; dexamethasone intraocular implants; and IV-MTX [45]. Treatment outcomes were evaluated at 12 weeks after initiation. The trial found that dexamethasone intraocular implants were significantly more effective than IVR and IV-MTX in improving BCVA and CFT resolution to baseline (Table 2). However, the use of dexamethasone intraocular implants was associated with an increased risk of elevated intraocular pressure. The study also reports that IV-MTX was the least effective treatment for reducing CFT and had the highest frequency of visual loss due to prolonged ME. Furthermore, at 24 weeks a higher proportion of eyes initially assigned to IV-MTX (55%) or IVR (37%) received additional non-assigned treatments—primarily dexamethasone implants or IVTA—compared to those assigned to dexamethasone implants (7%) [46].

Vitrectomy, which detaches and removes the ERM and relieves macular traction, may be beneficial in the treatment of UME. Additionally, macular retinal traction due to ERM formation is reported to contribute to the development of UME [3]. Because ERM formation is associated with a poor response to pharmacological treatment, preventing its onset may enhance therapeutic efficacy [47]. Vitrectomy is also reported to eliminate proinflammatory mediators accumulated in the vitreous body, potentially reducing dosages of corticosteroids or immunosuppressive agents [48, 49]. However, although vitrectomy has been shown to decrease the frequency of UME from 52% preoperatively to 37% postoperatively, recurrence remains common. Many cases require systemic administration of immunosuppressive agents or additional local therapy, such as STTA, following surgery [49].

#### In summary

UME may recur despite successful control of uveitic inflammation or may be refractory to corticosteroid therapy. Additionally, there are cases in which treatment is initially effective, but the side effects of corticosteroids hinder continued management. In Japan, cyclosporine (an immunosuppressive agent) and adalimumab (an anti–TNF agent) have recently been approved for insurance coverage for the treatment of noninfectious uveitis. However, despite the growing number of available therapies, UME persists in 44% of patients with uveitis, according to an OCT study [50]. Therefore, new agents, such as anti–IL-6 agents, are needed as alternative treatments for UME.

### Acute retinal necrosis

ARN is a disease caused by retinal infection with human herpes viruses, including HSV-1, HSV-2, and VZV [7, 8]. ARN is an uncommon condition, with an estimated annual incidence of 0.63 per million people in the United Kingdom [51]. In Japan, it accounts for approximately 1.7% of all diagnosed uveitis cases [52]. Although polymerase chain reaction (PCR) testing of intraocular fluid can confirm the presence of viral pathogens in ARN, diagnosis is primarily based on clinical evaluation—particularly fundoscopic findings. A hallmark feature of ARN is peripheral retinal necrosis that spreads circumferentially toward the posterior pole, often accompanied by occlusive vasculopathy, retinal arteritis, optic disc hyperemia, and vitreous opacities [53].

The visual prognosis is reported to be poor, with 48% of patients having a VA of 0.1 or less at six months after disease onset [51]. Several factors have been associated with unfavorable visual outcomes in ARN, including RD, necrotic involvement of zone 1, the extent of retinal tissue damage—such as optic nerve involvement and broad circumferencial necrosis—VZV etiology, and a high viral load, specifically defined as more than 5.0 × 10⁶ copies of VZV DNA in the AH [9, 54–58]. Although VZV-associated ARN has been traditionally considered to carry a poorer prognosis than HSV-associated ARN [57], recent meta-analytic findings do not support a significant difference in visual outcomes between the two [59].

Despite the poor visual prognosis associated with ARN, the absence of a standardized treatment protocol is partly attributable to the rarity of the condition, which makes large-scale clinical trials difficult to conduct. In general, treatment for ARN includes antiviral agents such as acyclovir, systemic corticosteroids, and antiplatelet therapy [55].

#### Findings and imaging characteristics of acute retinal necrosis

**Fig. 2:**
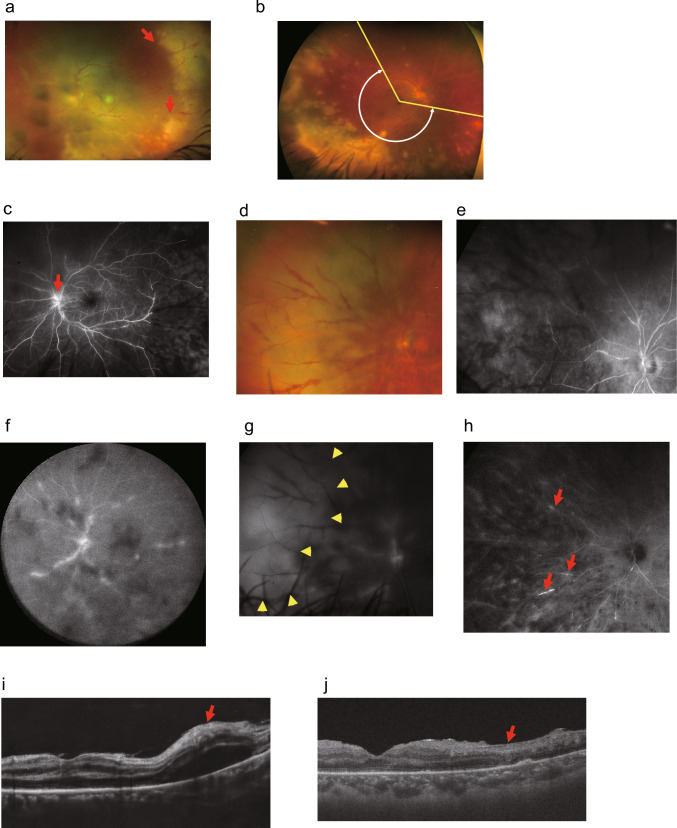
Multimodal imaging in patients with acute retinal necrosis (ARN).**a** Ultra-widefield fundus imaging shows yellow-white necrotizing retinitis in the peripheral temporal retina, with flame-shaped hemorrhages along the retinal arterioles and venules within the lesion (red arrows). **b** Measurement of extent of necrotizing retinitis in patients with ARN using ultra-widefield fundus images. To quantify the circumferential extent of necrotizing retinitis on ultra-widefield fundus images, two meridional lines (yellow lines) were drawn from the macula to both ends of necrotic lesions. Subsequently, the span (white circumferential line) between the meridians was measured to define the extent of the necrotizing lesions. **c** Fluorescein angiography reveals optic disc leakage and vascular occlusion in the temporal retina. **d** Color fundus photograph showing yellow-white necrotizing retinitis in the peripheral nasal retina. **e** Early-phase fluorescein angiography reveals dye leakage and vascular dropout in the nasal retina, with corresponding areas of choroidal hypofluorescence consistent with necrotizing retinitis. **f** Late-phase fluorescein angiography demonstrates granular or beaded dye leakage along the retinal arteries. **g** Late-phase fluorescein angiography shows choroidal hyperfluorescence in areas corresponding to necrotizing retinitis (outlined by yellow arrowheads). **h** Late-phase indocyanine green angiography reveals delayed choroidal filling and hyperfluorescence at sites of retinal arteritis (red arrows). **i** OCT image at an early stage of ARN showing hyperreflectivity and thickening of the inner retinal layers, along with serous retinal detachment (red arrows). **h** OCT image two months after onset shows destruction of the full-thickness retina. All images have been modified and reprinted with permission from reference [168]

Patients with ARN typically present with sudden, unilateral vision loss. At initial examination, visual decline is most often attributed to vitreous haze and other inflammatory manifestations associated with panuveitis. The presence of characteristic necrotic lesions—predominantly located in the peripheral retina—is a defining feature of the disease (Fig. 2a) [53]. Flame-shaped hemorrhage is frequently observed within these lesions (Fig. 2a, red arrows). In the early phase, optic disc hyperemia and swelling may occur due to optic neuritis (Fig. 2a). Accordingly, careful evaluation of the peripheral retina is critical, as these lesions tend to progress centrally toward the macula.

However, accurate assessment of peripheral necrotic changes at initial presentation can be hindered by media opacities, such as dense vitritis. Notably, poor VA at presentation has been identified as a significant risk factor for long-term visual impairment [60, 61]. The extent of necrotizing retinitis in ARN is sometimes described using a zonal classification system originally proposed by Holland et al. for cytomegalovirus retinitis [62], which divides the retina into zones 1, 2, and 3, from the posterior pole outward. A multicenter retrospective study conducted in Japan evaluating treatment efficacy in patients with ARN found that poor visual outcomes were significantly associated with posterior pole involvement (zone 1) and optic nerve damage [54].

Recent studies highlight the potential utility of ultra-widefield (UWF) imaging in detecting peripheral white necrotic lesions, even in the presence of vitreous opacities [63]. Due to its ability to capture a broad retinal area in a single image, UWF imaging is increasingly used to evaluate peripheral retinal pathology and vascular abnormalities in various ocular diseases, including ARN [64–66]. Using UWF fundus images obtained at initial presentation, we measured the area of necrotic lesions and demonstrated that a wider extent of peripheral involvement correlated with poorer visual outcomes one year after onset (Fig. 2b) [67]. In addition, UWF imaging enables longitudinal follow-up of peripheral retinal lesions. Thus, UWF imaging may be a valuable tool for predicting visual prognosis in ARN.

Fundus autofluorescence (FAF) imaging shows hyperautofluorescence in areas of active necrotizing retinitis. High-contrast autofluorescence patterns are reported to delineate the borders of disease activity and are useful for monitoring disease progression [68]. Fluorescein angiography (FA) in early optic nerve involvement typically reveals leakage and hyperfluorescence from the optic disc (Fig. 2c). In areas of necrotizing retinitis (Fig. 2d), FA may reveal vascular occlusion that predominantly affects retinal arteries, with interrupted perfusion and obstructive retinal vasculitis (Fig. 2e), loss of background choroidal fluorescence, and granular or beaded patterns of vascular leakage and non-perfusion (Fig. 2f). In typical cases, choroidal vasculature may also be involved, exhibiting early-phase hypofluorescence and late-phase hyperfluorescence consistent with inflammatory ischemia (Fig. 2g, yellow arrowheads). Indocyanine green angiography reveals delayed choroidal filling in the early phase, persisting into the late phase (Fig. 2h). Hyperfluorescence corresponding to areas of retinal arteritis is also observed in the late phase (Fig. 2h, red arrows).

OCT in early macular involvement typically reveals increased reflectivity and thickening of the inner retinal layers as initial findings (Fig. 2i, red arrow), reflecting inner retinal edema secondary to arterial occlusion. As the disease progresses, full-thickness retinal destruction is observed in areas corresponding to necrotic lesions (Fig. 2j, red arrow).

### Treatment

#### Antiviral and anti-inflammatory therapy

Antiviral treatment is typically initiated prior to virological confirmation by PCR, as ARN can progress rapidly. Systemic administration of antiviral agents not only treats the affected eye but also helps prevent involvement of the contralateral eye and brain due to their neurotropic nature of the causative viruses [69, 70]. Intravenous acyclovir remains the standard initial therapy for ARN, administered at an induction dose of 10 mg/kg three times daily for 7 days, followed by oral acyclovir 800 mg five times daily for 3–4 months [71]. In cases where VZV infection is suspected—given its tendency to cause more severe disease and lower responsiveness to standard-dose acyclovir—a higher dose of 15 mg/kg three times daily is recommended until the causative virus is identified [72]. Valacyclovir, a prodrug of acyclovir with enhanced oral bioavailability, may be considered as an alternative to intravenous acyclovir, even during the induction phase. Further, a prior retrospective study of 62 patients with ARN found no significant differences in VA, severe vision loss, or RD rates between patients treated with oral valacyclovir vs intravenous acyclovir, suggesting that oral valacyclovir is a clinically equivalent and more convenient outpatient alternative (Table 3) [73]. Combined therapy involving intravitreal injections of foscarnet (2.4 mg/0.1 mL weekly) or ganciclovir (2 mg/0.1 mL two or three times weekly) alongside systemic antiviral treatment is also reported to be effective in managing ARN [71, 74]. Flaxel et al. retrospectively demonstrated that patients with ARN who received combined systemic and intravitreal antiviral therapy achieved significantly superior visual outcomes, with a reduced incidence of severe vision loss and RD, compared with systemic therapy alone (Table 3) [75].

**Table 3:** Overview of previously published comparative clinical studies of treatment interventions in acute retinal necrosis

Reference, No.	Study type	Total number of eyes/patients	Mean age	Median follow-up period (months.)	Virus (VZV, %)	Treatment (number of patients)	The mean initial VA	The mean final VA (logMAR)	Incidence of RD
Baltinas J, et al. [73]	Retro-spective	68/62	48.4 ± 2.55	49.2	56	Intravenous acycrovir (n = 33) vs oral valacyclovir (n = 29)	1.01 vs 0.83 (NS)	1.14 vs 1.26 (NS)	62% vs 66% (NS)
Flaxel CJ, et al. [75]	Retro-spective	29/24	42.6 ± 23.3	44.0	11	Combination of systemic and intravitreal antiviral therapy (n = 12) vs systemic antiviral therapy alone (n = 12)	1.01 vs 0.68	0.59 vs 0.91 (at 6 months)	0.29 vs 0.74 (events per PY) (*P* = 0.03)
Hillenkamp J, et al. [80]	Retro-spective	30/27	58.0 ± 21.0	38.0	87	Prophylactic vitrectomy (n = 10) vs antiviral therapy (n = 20)	1.14 vs 1.28 (NS)	1.86 vs 2.14 (NS)	40% vs 90% (*P* = 0.007)
Iwahashi-Shima, et al. [54]	Retro-spective	104/104	51.2 ± 15.5	45.0	82	Prophylactic vitrectomy (n = 48) vs antiviral therapy (n = 56)	0.92 vs 0.64 (NS)	1.23 vs 0.92 (NS, at 1 year)	39.6% vs 69.6%

*VZV* varicella zoster virus, *VA* visual acuity, *logMAR* logarithmic minimum angle of resolution, *RD* retinal detachment, *NS* not significant, *PY* patient-years

A comprehensive review of 35 cases of ARN with primary treatment failure (PTF) to intravenous acyclovir showed that antiviral resistance, which is often linked to viral mutations in thymidine kinase or DNA polymerase genes, is a major contributor to PTF, particularly among immunocompromised individuals. True acyclovir resistance is uncommon, but suspected when new lesions develop after 10 days of therapy. Overall, this review highlights the importance of early and aggressive intervention, the use of combination regimens, including intravitreal foscanet or ganciclovir with systemic antivirals (intravenous acyclovir or oral valacyclovir), and the timely modification of therapy to minimize the risk of irreversible visual loss [76].

A recent retrospective study of patients with ARN reports that reduced steroid use—particularly in individuals with diabetes—increases the risk of RD due to inadequate control of inflammation [77]. These findings suggest that both anti-inflammatory and antiviral therapies help delay progression to RD, emphasizing the importance of early and intensive treatment, especially in high-risk patients. Conversely, a meta-analysis reviewing 34 studies involving 963 patients assessed outcomes associated with different antiviral strategies. Most cases were attributed to VZV (63%) or HSV (35%). Three primary treatment approaches were identified: oral antivirals, intravenous antivirals, and combined systemic and intravitreal antivirals. The pooled rates were 37% for visual improvement, 14% for recurrence, and 43% for RD. Although combination therapy demonstrated a trend toward better visual outcomes, the difference was not statistically significant [59].

#### Prophylactic vitreoretinal surgery for rhegmatogenous retinal detachment (RRD)

Some authors advocate prophylactic vitrectomy in ARN to remove inflammatory mediators and vitreoretinal traction, facilitate more precise laser photocoagulation, and permit silicone oil tamponade. However, the benefits of early or other prophylactic vitrectomy in eyes without RD remain controversial.

A recent study notes that only 2% of patients initially presented with RD; whereas previous reports demonstrated a 47–73% incidence of RD in ARN without surgical intervention [78–80]. Ishida et al. suggest that prophylactic vitrectomy helps prevent RRD in eyes with midperipheral retinal involvement [81]. A retrospective study reports that early vitrectomy combined with intravitreal acyclovir reduced the risk of RD compared to systemic therapy alone but did not significantly improve final VA (Table 3) [80]. Similarly, a multicenter retrospective study conducted in Japan evaluating treatment efficacy in patients with ARN found that prophylactic vitrectomy did not result in VA recovery (Table 3) [54]. A meta-analysis by Fan et al. demonstrates that prophylactic vitrectomy significantly reduced the risk of RRD; however, it was associated with poorer visual outcomes. This may be partly attributed to the potential macular toxicity of silicone oil. Although selection bias may be present, patients who underwent prophylactic vitrectomy typically had more extensive retinitis and more severe vitreous inflammation, which may have contributed to the poorer visual outcomes due to long-term complications such as chronic vitritis, proliferative vitreoretinopathy, ERM formation, macular ischemia, and optic atrophy [82].

#### In summary

ARN is a rare but vision-threatening condition that necessitates prompt diagnosis and treatment to prevent severe visual impairment. However, due to its low prevalence, large-scale data on the clinical features and management of ARN remain limited. Consolidation of data across multiple institutions through registries will enable large-scale analyses beyond the scope of individual case reports or small series. Notably, a study using the American Academy of Ophthalmology’s IRIS® Registry has already examined the association between initial treatment strategies, retinal detachment rates, and visual outcomes in patients with ARN [83]. In addition, a combined prospective and retrospective cohort study of ARN—the Japan-Acute Retinal Necrosis (J-ARN) Registry, has been established as a nationwide, multicenter database designed to collect comprehensive information on patient demographics, diagnostic approaches, treatment strategies, visual outcomes, and disease progression [84]. This registry aims to improve understanding of ARN’s etiology and clinical course, support the development of evidence-based treatment protocols, and enhance the accuracy of visual prognostication. The data collected are expected to inform clinical decision-making regarding antiviral therapy and vitrectomy and serve as a foundation for future investigations into optimized management and preventive strategies. Ultimately, this initiative may contribute to the development of novel treatment protocols that improve visual outcomes in patients with ARN.

### Vitreoretinal lymphoma

VRL is among the most prognostically unfavorable diseases in ophthalmology, with a reported 5-year survival rate ranging from 40% to 61.1% [11, 85]. VRL typically affects individuals aged 50 years and older, with a median age approximately 60 years [86]. A Japanese multicenter retrospective study reports a mean age of 63.4 years, with a range from 35 to 90 years [11]. Although several studies suggest a female predominance, with a female-to-male ratio of approximately 2:1.3, others have found no significant sex difference [87]. Even in cases initially confined to the eye, VRL demonstrates a high rate of CNS involvement, occurring in 60–85% of patients within a mean of 29 months [12]. The proportion of VRL cases has increased in recent years, likely reflecting improved diagnostic recognition and an aging population [88]. In British Columbia, Canada, the incidence increased from 0.023 to 0.047 per 100,000 population between the1990–1995 and the 2006–2010 periods [88]. Nationwide surveys conducted in Japan in 2002, 2009, and 2016 report that VRL accounted for a small but gradually increasing proportion of uveitis cases at university hospitals—1.0%, approximately 1.9%, and 2.5%, respectively [52, 89, 90]. Based on referral patterns, the annual incidence of VRL in Japan is conservatively estimated at 0.095 per 100,000 population [87].

VRL is often referred to as a ‘masquerade syndrome’ because its clinical findings, such as vitreous opacities, resemble those of uveitis. As a result, it is frequently misdiagnosed, with an average diagnostic delay of 10.9 months [11]. VRL is considered a subtype of PCNSL, with more than 90% of cases classified as diffuse large B-cell lymphoma (DLBCL) (28). Although no globally accepted treatment for VRL has been established, current therapeutic strategies include local ocular therapy, systemic therapy, or a combination of both approaches. Early detection is critical, and further elucidation of VRL’s pathophysiology may lead to the development of novel diagnostic methods and therapeutic targets. This section presents recent advances in the diagnosis and treatment of VRL.

#### Clinical findings of VRL

**Fig. 3:**
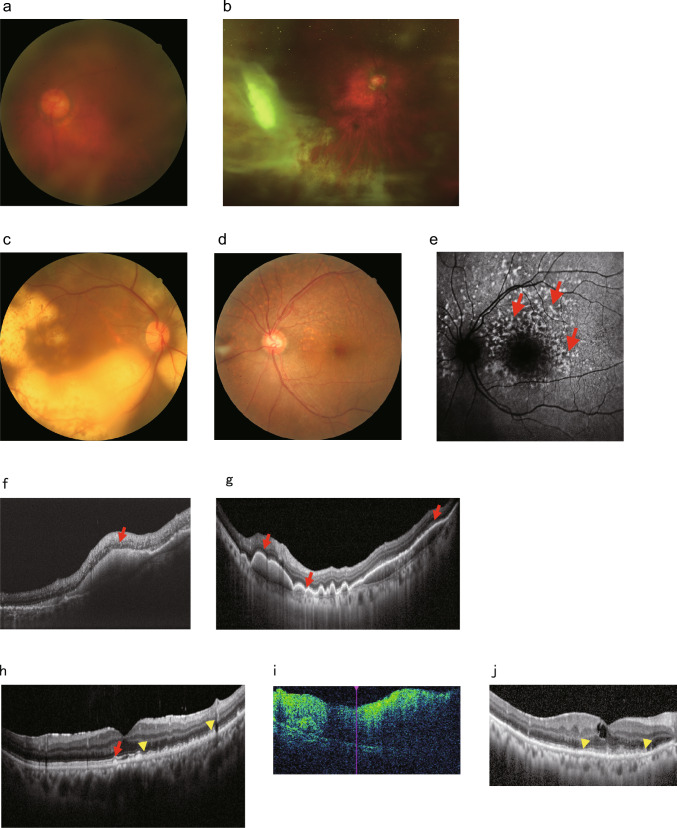
Multimodal imaging in patients with vitreoretinal lymphoma (VRL). **a**, **b** Fundus photographs showing string-like vitreous opacities in VRL. **b** Image acquired using ultra-widefield scanning laser ophthalmoscopy. **c**, **d** Fundus photographs of subretinal infiltrative lesions. **c** Subretinal yellowish-white elevated lesions with granular pigmentation is observed. **d** Small drusen-like subretinal lesions are present.**e** Fundus autofluorescence image showing a mixture of granular hyperautofluorescence and hypoautofluorescence areas corresponding to subretinal infiltrative lesions (red arrows). **f**–**j** OCT images of VRL. **f** Intraretinal infiltration (red arrows). **g** Subretinal infiltrates (red arrows). **h** Subretinal infiltration (red arrows) and thickening of the retinal pigment epithelium (between yellow arrowhead). **i** Intraretinal infiltration with complete disruption of retinal layer architecture. **j** Band-like subretinal deposits (between yellow arrowheads). All images have been modified and reprinted with permission from references [169, 170]

VRL presents bilaterally in approximately 50%–80% of cases. When vitreous opacities are the only clinical manifestation, distinguishing VRL from uveitis based on appearance alone is challenging. As vitreous opacities become more pronounced, characteristic features—described as veil-like or aurora borealis-like—are observed in over 90% of VRL cases, raising clinical suspicion for lymphoma (Fig. 3a and b) [11, 91]. In 50% to 57% of cases (Fig. 3c) [11, 92], ophthalmoscopy reveals subretinal infiltrative lesions located beneath the RPE and/or within the retina, appearing yellowish-white with granular pigmentation. These so-called leopard-skin-like lesions are typically multifocal and scattered, with smaller lesions resembling drusen. Retinal vasculitis occurs in approximately 10% of cases [11], and vascular occlusions, such as retinal vessel obstruction, may also be observed. It is also common for vitreous opacities and subretinal infiltrative lesions to coexist. Anterior uveitis is usually mild, and approximately 25% of patients exhibit small, granulomatous white keratic precipitates (KPs) [11]. However, the presence of iridocyclitis and KPs alone is insufficient to reliably distinguish VRL from other forms of uveitis.

Among multimodal imaging techniques, FAF is particularly useful for diagnosing VRL [93]. In the active phase, granular hyperautofluorescence and hypofluorescent may be observed in regions corresponding to subretinal infiltrative lesions (Fig. 3d and e, red arrows). In VRL, hyperautofluorescence is believed to result from dysfunction of the RPE due to sub-RPE infiltration by lymphoma cells or from the intrinsic autofluorescent properties of lymphoid cells. In contrast, hypoautofluorescence is thought to arise from blocked fluorescence due to infiltration of lymphoma cells into the RPE or from overlying atrophic RPE [94, 95].

OCT is also valuable for detecting retinal lesions in VRL. Characteristic OCT findings include hyperreflective subretinal infiltrates, hyperreflective inner retinal infiltration (Fig. 3f, red arrow), RPE undulations, clumps of vitreous cells, and sub-RPE infiltrates. Additionally, elevated lesions with hyperreflective material located between the RPE and Bruch’s membrane are often observed (Fig. 3g, red arrows) [96]. Guan et al. compared spectral-domain OCT findings from biopsy-confirmed primary vitreoretinal lymphoma (PVRL) patients and those with uveitis [97]. Findings them to be strongly suggestive of VRL, as compared to uveitis, included vitreous cells (93%), subretinal infiltration (51%), and diffuse RPE elevation (56%, Fig. 3h, between yellow arrowheads). Features specific to VRL included preretinal deposits (92.5%), full-thickness retinal infiltration (100%, Fig. 3i), band-shaped subretinal hyperreflective deposits (90%, Fig. 3j, between yellow arrowheads), and confluent RPE detachments (100%). Diagnostic criteria consisting of (1) two strongly suggestive findings and one specific finding, or (2) one strongly suggestive finding and two specific findings, achieved a sensitivity of 80% and specificity of 95%. A retrospective study of 95 patients with VRL identified sub-RPE infiltration and older age as predictors of shorter survival, whereas other clinical features and prophylactic chemotherapy were not significantly associated with prognosis [98].

#### Diagnosis

Cytology has been the gold standard for diagnosing VRL. The detection of tumor cells by cytology is universally regarded as the optimal criterion for initiating treatment. International diagnostic guidelines for VRL recommend performing diagnostic vitrectomy to obtain biopsies from the vitreous and/or subretinal lesions when VRL is suspected, particularly when hallmark findings such as vitreous opacities and subretinal lesions are present [93]. However, the diagnostic yield of vitreous biopsy is limited by factors such as small sample volume, cellular necrosis, and the fragility of VRL cells. Discontinuing systemic corticosteroid administration at least two weeks before surgery and using a cutter speed below 1500 rpm are recommended in the guidelines to improve sample yield and minimize cell damage [93]. Undiluted VH collected at the start of vitrectomy—before infusion begins—is typically used for cytological evaluation. In Japan, the Papanicolaou classification is commonly used, with classes IV and V considered diagnostic [87]. However, the detection rates of class IV or higher tumor cells in smear cytology remain relatively low (25–44.5%) due to limited sample volume [11, 99]. Recently, the cell block method using vitreous infusion fluid collected during vitrectomy has improved diagnostic yield by increasing the number of detectable tumor cells [100–102]. This method allows for both cytological and immunohistochemical analyses, thereby enhancing diagnostic accuracy. In cases with minimal vitreous haze and only subretinal infiltrates, direct biopsy of the subretinal lesion is reported to be effective [103]. According to data from an international clinical registry for B-cell VRL, in real-world clinical practice, 80% of patients were diagnosed with VRL based on cytological findings, while the remaining 20% were diagnosed using other methods, including clinical information [92].

#### Ancillary diagnostic tests

Since cytology alone is often insufficient to confirm a diagnosis of VRL, ancillary tests are used alongside clinical findings to achieve a comprehensive diagnosis. These tests include IL-10/IL-6 cytokine analysis, detection of immunoglobulin heavy chain (IgH) monoclonality, identification of skewed κ/λ light chain ratios in B-cell populations via flow cytometry, and gene mutation analyses. A comparative study evaluating the diagnostic sensitivities of various modalities—such as cytology, flow cytometry, *myeloid differentiation factor 88* (*MYD88*) *L265P* and *CD79B* mutation analyses, IL-10/IL-6 ratio, PCR for IgH and immunoglobulin kappa light chain gene rearrangements, and imaging findings—demonstrate that an IL-10/IL-6 ratio ≥ 1 yielded the highest diagnostic sensitivity for PVRL comparable to that of flow cytometry of vitreous samples and PCR for IgH monoclonal gene rearrangements [13]. In contrast, the sensitivities of *MYD88 L265P* and *CD79B* mutation analyses were lower than those of other ancillary diagnostic tests, such as flow cytometry and PCR-based detection of IgH monoclonality. Although ancillary diagnostic tests can support the diagnosis of VRL, they cannot serve as standalone diagnostic tools. For example, cytokine concentrations in the VH may be influenced by prior treatment with corticosteroids or immunosuppressive agents, potentially leading to misinterpretation of results. Additionally, there are reported cases of VRL in which the IL-10/IL-6 ratio was less than 1 or IL-10 levels were undetectable, further highlighting the limitations of relying solely on cytokine analysis for diagnosis. Therefore, results from ancillary diagnostic tests should be interpreted in conjunction with clinical findings and other diagnostic modalities. Tanaka et al. propose that a diagnosis of VRL is sufficiently supported either by the presence of malignant cells on cytology or by positive results in at least two of the following four vitreous-based tests: malignant cytology or atypical cells with uncertain significance, IL-10/IL-6 ratio ≥ 1, IgH gene rearrangement, and flow cytometric detection of clonal B cells [104].

#### Gene mutation analysis

Representative mutations in VRL include *MYD88 L265P* and *CD79B Y196*. MYD88 encodes as an adaptor protein involved in Toll/IL-1 receptor signaling that activates the nuclear factor κ-light-chain-enhancer of activated B cells (NF-κB) pathway. Constitutive activation of NF-κB plays a key role in the pathogenesis of activated B-cell–like diffuse large B-cell lymphoma (ABC DLBCL), a subtype associated with poor prognosis and comprising the majority of primary central nervous system lymphoma (PCNSL) cases [105]. The *MYD88 L265P* mutation is the most frequently observed genetic alteration in VRL, detected in 40–82% of cases using PCR, allele-specific PCR with Sanger sequencing, or digital PCR [106–111], and is considered a strong diagnostic marker, as endorsed by international diagnostic guidelines for VRL [93]. *CD79B* encodes the Igβ component of the B-cell receptor (BCR) complex. Mutations within its immunoreceptor tyrosine-based activation motif domain can result in sustained BCR signaling, ultimately leading to NF-κB activation. The *CD79B Y196* mutation, found in 35%–55% of cases [111–113], has been associated with early CNS involvement [111]. Genetic profiling of DLBCL has identified the MCD subtype, defined by co-occurrence of *MYD88* and *CD79B* mutations. This subtype shares genetic features with the ABC phenotype and has shown a favorable response to ibrutinib, a first-generation of Bruton’s tyrosine kinase (BTK) inhibitor, in relapsed or refractory ABC DLBCL and PCNSL with an ABC-like profile [114].

A comprehensive, integrated DNA/RNA next-generation sequencing (NGS) assay targeting hundreds of genes has demonstrated significant clinical utility in hematologic malignancies by enabling the detection of various clinically relevant alterations, including somatic mutations, gene fusions, and germline variants. This supports its application in diagnosis, therapeutic decision-making, and prognostic evaluation [115]. In Japan, a gene panel for hematological malignancies has been in clinical use since its validation in March 2025. Building on these advances, panel-based NGS allows for the simultaneous analysis of multiple genetic mutations, facilitating the classification of malignancies based on their genetic profiles. Accordingly, several recent studies have explored its application in VRL. Amplicon-based NGS of VH from patients with VRL revealed a recurrent mutational profile, including *MYD88* (74%), *PIM1* (71%), *CD79B* (55%), *IGLL5* (52%), *TBL1XR1* (48%), *ETV6* (45%), and 9p21/*CDKN2A* alterations (75%), closely resembling the MCD subtype of DLBCL. This study also demonstrates that mutation analysis using circulating tumor DNA (ctDNA) is a sensitive and specific diagnostic approach for VRL [112]. Furthermore, recent genomic studies employing whole-exome sequencing or amplicon-based NGS show that *ETV6* deletions and *PRDM1* gene alterations are associated with CNS dissemination [116]. ETV6, a member of the ETS transcription factor family, plays a key role in hematopoiesis and is implicated in various hematologic malignancies, including DLBCL. Although the biological significance of *ETV6* loss in PVRL remains to be fully elucidated, its protein expression has been shown to inversely correlate with that of BIRC5 (survivin), a known regulator of cell survival. Deletion of *ETV6* disrupts its tumor suppressor function [117]. PRDM1, which also functions as a tumor suppressor in both B- and T-cell lymphomas, is reported to contribute to the poor prognosis of ABC DLBCL when inactivated. This occurs through mechanisms such as ABC-specific genetic aberrations, impaired p53 signaling, *Myc* overexpression, and gene expression changes indicating reduced plasmacytic differentiation and enhanced BCR signaling [118].

In addition, one study examined the genetic mutation profiles of VRL and uveitis patients using NGS with a 446-gene panel. The results indicate that ctDNA in AH and VH samples was highly sensitive for diagnosing VRL, showing significant differences in mutation frequency and somatic mutations between VRL and uveitis patients. This approach achieved a sensitivity of 100%, specificity of 66.7%, and an overall test efficiency of 91.3%. Molecular classification of VRL revealed frequent mutations in genes such as *PIM1* (91%), *MYD88* (77%), and *CD79B* (50%), with most patients classified as the MCD subtype [119]. The study also demonstrates strong concordance between AH and VH samples, with ctDNA allele frequency changes correlating with IL-10 levels, which serve as biomarkers for treatment response [113]. Additionally, comparisons with PCNSL patients revealed differences in mutation profiles and ibrutinib treatment efficacy, further supporting the utility of AH for VRL diagnosis and monitoring.

#### Comprehensive analysis of intraocular fluids in VRL

Given that measuring the IL-10 and IL-6 levels has proven to be a useful diagnostic tool with high sensitivity and specificity [93, 104], the analysis of intraocular fluid is considered highly effective for identifying novel diagnostic markers to differentiate VRL from uveitis and for discovering new therapeutic targets through the elucidation of disease pathogenesis. Several groups report comprehensive analyses of intraocular cytokines. A detailed analysis of T helper (Th)1/Th2 cytokines in intraocular fluid samples from patients with VRL, uveitis, and non-inflammatory retinal detachment demonstrates that the IL-10/IL-6 and IL-10/interferon gamma ratios are particularly informative for distinguishing VRL from uveitis and serve as useful tools for early diagnosis and therapeutic monitoring [120]. In addition, our findings indicate that vitreous cytokine and soluble cytokine receptors’ profiles differ between B-cell VRL and uveitis. Specific markers—such as soluble VEGF receptors 1 and 2 and soluble IL-2 receptor α—may offer potential for differential diagnosis and prediction of retinal or subretinal infiltration in VRL [121]. An analysis of 13 regulatory cytokines demonstrated that elevated intravitreal IL-35 levels are associated with poor prognosis in patients with B-cell VRL, suggesting its potential as a prognostic biomarker [122]. Furthermore, a combined approach employing flow cytometry and cytokine profiling has enabled accurate diagnosis of PVRL by detecting a distinct diagnostic signature comprising cellular parameters (CD19⁺ B cells, aberrant surface immunoglobulin light chain expression) and cytokine parameters (IL-10/IL-6 ratio > 1; elevated IL-10, IL-1 receptor antagonist, monocyte chemotactic protein-1, and macrophage inflammatory protein-1β), even in cases with minimal cellular infiltration [123]. Recent studies employing advanced artificial intelligence demonstrate that machine learning algorithms—particularly the random forest model—can accurately predict the diagnosis of 12 intraocular diseases by analyzing 28 cytokines and other soluble factors in the VH. In the case of VRL, key mediators identified include IL-10, granzyme A, and IL-6 [124]. Furthermore, advances in proteomic technology have enabled the identification of hundreds to thousands of proteins within a single sample and allow for the quantitative comparison of protein expression across samples based on peptide fragment detection intensities. A comprehensive proteomic analysis of VH, performed using liquid chromatography and mass spectrometry, identified differentially expressed proteins and proteasome-related pathway alterations in VRL, including upregulated proteins such as PSAT1, YWHAG, and components of the 20S/26S proteasome complex [125].

MicroRNAs (miRNAs) are small non-coding RNAs that regulate gene expression by binding to target messenger RNA (mRNA) sequences, thereby suppressing translation and promoting mRNA degradation. Several studies have identified distinct miRNA expression profiles in patients with VRL compared to those with uveitis, suggesting potential candidates for differential diagnostic biomarkers. Tuo et al. report unique miRNA signatures in the vitreous of patients with PVRL and uveitis, identifying elevated levels of miRNA-484, miRNA-197, and miRNA-132 in VRL, while miRNA-155, miRNA-200c, and miRNA-22 were elevated in uveitis. Among 168 miRNAs examined, a significant increase in miRNA-155 in uveitis cases was proposed as a potential marker for distinguishing between the two diseases [126]. In contrast, another group reports that upregulation of miRNA-92, miRNA-19b, and miRNA-21 in the vitreous of patients with VRL, each demonstrating an area under the curve greater than 0.8. Furthermore, a comprehensive analysis of thousands of miRNAs revealed that aberrant miRNA expression was significantly associated with extracellular matrix–receptor interaction and other cancer-related pathways in VRL. Notably, miRNA-326 was identified as a driver of B-cell proliferation, miRNA-1236-3p was found to correlate with IL-10 levels, and miRNA-6513-3p and miRNA-361-3p served as discriminators from uveitis, highlighting their potential utility as diagnostic biomarkers for VRL [127].

#### Treatment

Currently, there are no standardized treatment guidelines for VRL, including PVRL, and its management requires a multidisciplinary approach involving ophthalmologists, hematologists, neurosurgeons, and radiologists. Ophthalmologists are primarily responsible for the diagnosis, treatment, and monitoring of intraocular involvement, whereas the other specialists manage CNS and systemic lesions.

Treatment strategies vary internationally and across institutions. Local therapies, such as IV-MTX, intravitreal rituximab (IV-RTX), and ocular radiotherapy, are commonly employed for intraocular diseases. Systemic therapies include high-dose methotrexate (HD-MTX)-based chemotherapy, intrathecal methotrexate, whole-brain radiotherapy (WBRT), and autologous stem cell transplantation (ASCT).

The International Primary CNS Lymphoma Collaborative Group introduced guidelines in 2011 and updated them in 2018, recommending IV-MTX as initial therapy for unilateral cases, with local treatments reserved for recurrence when the CNS is uninvolved [128, 129]. In contrast, bilateral cases may necessitate systemic chemotherapy in combination with local therapy. The 2019 guidelines by the British Neuro-Oncology Society recommend systemic HD-MTX and rituximab (RTX), followed by extensive radiotherapy, irrespective of CNS involvement [130]. Meanwhile, the French LOC network’s 2021 recommendations support the use of HD-MTX combined with low-dose ocular radiation for patients in stable general health, and IV-MTX for rapid intraocular disease control or in those with poor systemic status [131]. Despite these national differences, systemic chemotherapy is generally advised for patients with good performance status.

### Local therapy

#### Intravitreal MTX

The first cohort study on IV-MTX for VRL, conducted in the mid-1990s, reported that weekly IV-MTX injections at a dose of 400 µg were both safe and effective for the treatment of VRL [132]. A 10-year follow-up study by Frenkel et al., employing a structured 12-months regimen, demonstrates clinical remission in all treated eyes; however, keratopathy and cataract formation were frequently observed as adverse effects [133]. Despite achieving local disease control, more than 60% of the patients reportedly died from CNS or systemic involvement within a median of 17 months following treatment with IV-MTX monotherapy [133]. In addition, a multicenter study on ocular involvement in PCNSL indicates that ocular therapy improved local control but did not affect relapse rates or overall survival (OS) [134]. Furthermore, long-term data further suggest that most patients treated exclusively with IV-MTX eventually develop CNS involvement [135]. In some cases, persistent subretinal infiltrates without scarring raise doubts about the completeness of remission. Such cases often require additional therapies including IV-RTX or local radiotherapy, in conjunction with serial monitoring of IL-10/IL-6 ratios to confirm disease control [136].

#### Intravitreal RTX

RTX is a monoclonal antibody that targets CD20, a surface antigen commonly expressed on B cells in PCNSL and VRL. RTX induces B-cell depletion through antibody-dependent cellular cytotoxicity, complement activation, and apoptosis [137]. A prospective study evaluated the long-term outcomes of IV-RTX injections in 13 women (20 eyes) with CD20-positive PVRL who had discontinued IV-MTX due to corneal toxicity. A single course of weekly IV-RTX injections improved both anterior and posterior segment inflammation; however, 55% of eyes experienced recurrence, requiring additional treatment [138].

In a separate retrospective study involving 48 eyes from 34 patients with VRL, most eyes received IV-RTX for newly diagnosed or progressive disease. Complete remission (CR) was achieved in 64.6% of eyes after a median of three injections, and partial remission (PR) in 22.9%. Although ocular complications were reported in some cases, significant VA loss was rare [139]. Overall, these findings suggest that IV-RTX is a generally safe and effective treatment option for VRL, particularly in patients who are intolerant to MTX. Additionally, a gradual reduction in the therapeutic effect of RTX has been observed over time, consistent with resistance mechanisms identified in systemic lymphoma, such as CD20 alterations, impaired apoptotic signaling, complement regulation, and reduced immune-mediated cytotoxicity [137].

#### Ocular irradiation

Ocular irradiation, a traditional treatment modality for VRL, has demonstrated efficacy in inducing remission, with one study reporting a CR rate of 87% in patients with PVRL [140]. However, nearly half of the patients experienced relapse shortly thereafter, and the 2-year recurrence-free survival rate was only 58%. A recent retrospective study found that combining ocular irradiation with subsequent systemic MTX therapy helped prevent CNS involvement and improve clinical outcomes. Notably, neither the median progression-free survival (PFS) nor the OS had been reached during a median follow-up period of 68 months [141]. Although radiotherapy can be effective, its use is limited by the potential for adverse effects such as radiation retinopathy and neurotoxicity—particularly at doses exceeding 35 Gy—although complications have also been reported at lower doses. In Japan, ocular irradiation is generally reserved for severe cases that are resistant to IV-MTX or for patients with poor general health who are unable to undergo systemic therapy.

#### Systemic therapy

There is ongoing debate regarding the necessity of systemic therapy to prevent CNS dissemination in PVRL. A retrospective French study (2011–2018) demonstrates that HD-MTX-based chemotherapy reduced the risk of brain relapse in patients with isolated PVRL; however, frequent ocular relapse underscored the need for improved local control. Median OS was 75 months, with PFS, ocular relapse-free survival, and CNS relapse-free survival reported as 18, 29, and 73 months, respectively (Table 4) [142]. A retrospective study from the Mayo Clinic (1990–2018) found that patients with PVRL, VRL concurrent with CNS or systemic disease, and secondary VRL experienced improved outcomes with combined systemic and intraocular therapy. This approach reduced CNS relapse and improved OS—particularly after 2000—supporting the use of combination treatment to prevent CNS progression [143]. Another retrospective French study (2008–2019), which included 38 immunocompetent patients with refractory VRL treated with high-dose chemotherapy and ASCT, demonstrates acceptable safety, although CNS relapse remained common. Median PFS, brain relapse-free survival, and OS were 96, 113, and 92 months, respectively (Table 4) [144]. Two prospective studies have evaluated combination therapies aimed at preventing CNS relapse in PVRL. In the first, IV-MTX followed by systemic HD-MTX chemotherapy achieved CR in all 10 patients, with a 2-year CNS lymphoma-free survival rate of 58.3%, compared to 37.5% in those receiving IV-MTX monotherapy [145]. The second study employed a more intensive regimen—including IV-MTX, HD-MTX based chemotherapy, high-dose cytarabine, and reduced-dose WBRT—resulting in 4-year PFS and OS rates of 74.9% and 86.3%, respectively [136]. Furthermore, a meta-analysis of 37 studies involving 801 patients demonstrates that systemic therapy and combined treatment strategies for VRL achieve high remission rates and OS [146]. Collectively, these findings suggest that combination approaches may reduce the risk of CNS relapse and improve long-term outcomes in patients with PVRL.

**Table 4:** Systemic treatment outcomes in refractory vitreoretinal lymphoma

Reference, No.	Study type	Total VRL Patients.	Median age (range)	Systemic therapy	Median follow-up period (months.)	CR (%)	Median PFS (months.)	Median OS (months.)	CNS relapse rate(%)	5-year OS rate (%)	Death (%)
Lam M, et al. [136]	Retro-spective	59	70 (39–88)	HD-MTX	61	70	18	75	37	67	34
Mainguy A, et al. [138]	Retro-spective	38	59.5 (40–71)	HCT-ASCT	79	87	96	92	45	71	40
Soussain C, et al. [146]	Pro-spective	14	70 (52–81)	Ibrutinib	25.7	74	22.7	Not reached	7.1	Not reached	14.3
Baron M, et al.[152]	Retro-spective	21	75 (35–90)	Temozolomide	42	71	12	Not reached	23.8	Not reached	28.6

VRL vitreoretinal lymphoma, CR complete response, PFS progression free survival, OS overall survival, HD-MTX high-dose methotrexate-based chemotherapy, HCT-ASCT high-dose chemotherapy with autologous stem cell transplantation, CNS central nervous system

On the other hand, a retrospective study of 83 immunocompetent patients conducted by the International Primary CNS Lymphoma Collaborative Group reports that local treatment alone—such as IV-MTX or ocular radiotherapy—was associated with relapse rates, PFS, and OS comparable to those observed with systemic or combined therapies, suggesting that local therapy reduces toxicity without compromising disease control [147]. In a multicenter European retrospective study of 78 patients with PVRL without CNS involvement at diagnosis, systemic chemotherapy did not significantly reduce the risk of CNS progression compared to local treatment and was associated with more severe adverse effects. CNS involvement developed in 36% of patients, with similar incidence rates across treatment groups; moreover, systemic therapy was associated with increased toxicity, including acute renal failure [148]. A meta-analysis of 28 studies comprising 476 patients assessed various treatments for PVRL. Ocular therapies, such as IV-MTX or radiotherapy, were associated with lower risk of CNS progression and ocular relapse compared to systemic treatment. No survival benefit was identified with the addition of systemic therapy, suggesting that ocular treatment alone is sufficient in many cases [85]. Furthermore, a separate meta-analysis evaluating PVRL treatment found that the combined approach of systemic and local therapy resulted in the longest median survival; however, this benefit diminished after adjustment for lead-time bias associated with CNS progression [149].

HD-MTX-based chemotherapy and ASCT are often not feasible in elderly patients or those with poor general performance status due to systemic adverse effects, such as leukoencephalopathy and renal failure, which may limit the number of patients eligible for systemic therapy. While PCNSL is frequently associated with a gene expression profile resembling the ABC/MCD subtype of DLBCL, VRL has more often been reported to exhibit features characteristic of the germinal center B-cell–like subtype, associated with a relatively better prognosis than the ABC subtype [150]. As VRL may involve a higher proportion of biologically less aggressive cases, the prognostic benefits of systemic therapy may be limited in this population. In the future, genomic profiling may facilitate the identification of patients likely to benefit from systemic therapy, as well as those for whom local ocular treatment alone may be sufficient. Furthermore, novel therapeutic agents are emerging that may be suitable even for elderly patients who are ineligible for conventional systemic regimens.

### Emerging therapies

#### BTK inhibitors

BTK is a central component of BCR signaling and is essential for B-cell proliferation. Its selective expression in B cells makes it an effective therapeutic target [151]. Dysregulation of this pathway contributes to B-cell malignancies, and BTK inhibitors have become standard treatments in several of these diseases [152]. These agents inhibit BCR signaling, leading to reduced activation of NF-κB, a critical driver of B-cell lymphoma proliferation. By suppressing NF-κB activity, BTK inhibitors limit lymphoma cell growth and promote apoptosis in malignant B cells [151]. Additionally, BTK inhibitors are capable of crossing the blood–brain barrier (BBB), suggesting their therapeutic potential as agents for CNSL [153].

A phase II multicenter study evaluated ibrutinib—a first generation covalent BTK inhibitor—as monotherapy in patients with relapsed or refractory DLBCL involving the CNS or eye. Among 52 patients, the two-month disease control rate was 70% in evaluable cases. Median PFS and OS were 4.8 and 19.2 months, respectively (Table 4). Ibrutinib demonstrated clinical activity in the brain, cerebrospinal fluid, and intraocular space, with tolerable toxicity. Clinical responses were observed regardless of mutations in the BCR signaling pathway [154], supporting further investigation of ibrutinib in combination with MTX-based chemotherapy as a first-line treatment option. Prolonged use of covalent BTK inhibitors may lead to acquired resistance due to mutations in BTK (C481) or its downstream target PLCγ2 [155]. To enhance tolerability and reduce the off-target effects seen with earlier agents such as ibrutinib, second-generation covalent BTK inhibitors with greater selectivity—such as tirabrutinib—have been developed. These agents have shown promise in both treatment-naïve and relapsed/refractory PCNSL [155, 156]. A phase I/II study assessed the safety, pharmacokinetics, and efficacy of tirabrutinib in 44 patients with relapsed or refractory PCNSL, including three patients with intraocular involvement. Patients received 320 mg or 480 mg once daily, including a fasting cohort. The overall response rate was 64%, with better outcomes observed at the 480 mg dose. Median PFS ranged from 2.1 to 11.1 months, depending on dosage and treatment conditions. All patients with concurrent VRL (3 of 3) achieved PR. Common grade ≥3 adverse events included neutropenia, lymphopenia, and erythema multiforme. Responses were observed irrespective of *CARD11*, *MYD88*, or *CD79B* mutation status, supporting tirabrutinib’s broad therapeutic potential [157]. In Japan, tirabrutinib is approved for relapsed/refractory (R/R) CNSL and is currently under clinical investigation for PVRL due to its potential to prevent CNS progression and improve OS [87, 158].

#### Temozolomide

Temozolomide (TMZ), an orally administered alkylating agent with good BBB penetration, induces DNA mismatches through methylation, thereby triggering mismatch repair–mediated G_2_/M arrest and tumor cell apoptosis [159]. Its clinical utility has been retrospectively evaluated in 21 patients with R/R or treatment-ineligible PVRL, including elderly individuals[160]. This cohort, primarily composed of older and heavily pretreated patients, received monthly TMZ, achieving an overall response rate of 81% and a CR rate of 71%, with durable remissions and manageable toxicity. The median PFS was 12 months, while OS had not been reached at a median follow-up of 42 months (Table 4). TMZ was well tolerated compared to existing therapies, suggesting it is a promising option for elderly or R/R PVRL patients, particularly when intensive standard treatments are not feasible. According to the 2021 guidelines of the French LOC network, oral TMZ, ocular radiotherapy (30 Gy), or IV-MTX is recommended for patients with poor general condition [131]. Similarly, at the Mayo Clinic, systemic therapies including MYX, RTX, and TMZ have, in bilateral cases been combined with IV-MTX and/or IV-RTX [129]. However, it is important to note that in glioblastoma multiforme, resistance to TMZ has primarily been attributed to DNA repair mechanisms that eliminate O⁶-methylguanine adducts, with additional contributions from miRNAs, drug efflux transporters, gap junction activity, glioma stem cells, and autophagy [159].

#### Lenalidomide plus rituximab

Lenalidomide, a second-generation immunomodulatory drug, is more potent and less toxic than thalidomide. It exerts antitumor effects by inhibiting angiogenesis, inducing apoptosis and cell cycle arrest, and disrupting tumor–stromal interactions [161]. Immunologically, it enhances T-cell and natural killer cell activity, suppresses regulatory T cells, and downregulates immune checkpoints such as PD-1 and PD-L1. These mechanisms support its efficacy in multiple myeloma and other hematologic malignancies [161]. A prospective phase II study investigated the therapeutic potential of RTX plus lenalidomide (R2) in patients with R/R PVRL and PCNSL [162]. Among the 50 enrolled patients, 11 had PVRL. The overall response rate after induction therapy was 35.6%, with 29% achieving complete or unconfirmed complete responses. The treatment was generally well tolerated, and a higher baseline CD4/CD8 ratio was associated with longer PFS. These results indicate that the R2 regimen may offer clinical benefits in managing PVRL, particularly for patients with limited treatment options, and support further evaluation of R2 in combination therapies for VRLs.

#### Chimeric antigen receptor T-cell therapy

Anti-CD19 chimeric antigen receptor (CAR) T-cell therapy, which employs genetically engineered T lymphocytes, has significantly advanced the treatment of aggressive B-cell lymphomas, particularly DLBCL [163]. Clinical trials demonstrate strong response rates and improved survival in CNSL [164], leading to its approval as a second- and third-line therapy. However, its use is often limited by serious side effects, notably cytokine release syndrome (CRS) and immune effector cell–associated neurotoxicity syndrome (ICANS), with the latter affecting up to 60% of patients [165]. Although CNSL patients were typically excluded from early trials, CAR T-cell therapy has been permitted in France for R/R CNSL since 2020. Given the shared antigen targets, FDA-approved anti-CD19 CAR T-cell therapies, such as axicabtagene ciloleucel, may be applicable to PVRL, though their use remains restricted to R/R cases due to significant toxicity risks, including CRS, ICANS, infections, and cytopenia [166]. A case report describes a 59-year-old man with systemic DLBCL who developed VRL recurrence following CD19 CAR T-cell therapy, despite achieving systemic remission. This suggests limited intraocular trafficking of CAR T-cells and underscores the need for ophthalmic surveillance following treatment [167].

#### In summary

The optimal management of PVRL remains controversial, and treatment strategies should be tailored to individual patients based on factors such as the laterality of ocular involvement, CNS involvement, and overall physical condition. Further research is warranted to establish standardized therapeutic approaches. Although the majority of VRL cases are classified as DLBCL, DLBCL itself represents a biologically heterogeneous entity characterized by diverse genetic alterations. Accordingly, VRL is among the conditions for which advanced diagnostic modalities, including gene panel testing, may offer clinical utility. The integration of emerging technologies, such as genetic profiling, proteomics, and single-cell RNA sequencing, has the potential to provide a more comprehensive understanding of the molecular pathogenesis of VRL. Furthermore, international, multicenter prospective studies and randomized controlled trials are essential for the development of evidence-based diagnostic criteria and standardized treatment regimens for this rare and challenging disease.

#### Conclusions and future directions

This review focused on the three most commonly refractory ocular inflammatory diseases: UME, ARN, and VRL. These conditions remain major diagnostic and therapeutic challenges, with limited standardized treatment strategies. Recent advances in imaging, molecular diagnostics, and biologic therapies have improved disease recognition and management; however, robust clinical evidence is still lacking. Future progress will depend on multicenter collaboration and precision medicine approaches to establish evidence-based guidelines and improve long-term outcomes. We hope that these efforts will ultimately achieve meaningful improvements in visual function or survival for patients affected by these three refractory ocular inflammatory diseases.
